# Microstructure and Mechanical Characterization of Novel Al_2_O_3_–(NiAl–Al_2_O_3_) Composites Fabricated via Pulse Plasma Sintering

**DOI:** 10.3390/ma16114136

**Published:** 2023-06-01

**Authors:** Justyna Zygmuntowicz, Katarzyna Konopka, Marek Krasnowski, Paulina Piotrkiewicz, Marcin Wachowski, Radosław Żurowski, Konrad Cymerman, Krzysztof Kulikowski, Robert Sobiecki

**Affiliations:** 1 Faculty of Materials Science and Engineering, Warsaw University of Technology, 141 Woloska St., 02-507 Warsaw, Poland; katarzyna.konopka@pw.edu.pl (K.K.); marek.krasnowski@pw.edu.pl (M.K.); paulina.piotrkiewicz.dokt@pw.edu.pl (P.P.); konrad.cymerman.dokt@pw.edu.pl (K.C.); krzysztof.kulikowski@pw.edu.pl (K.K.); robert.sobiecki@pw.edu.pl (R.S.); 2 Faculty of Mechanical Engineering, Military University of Technology, 2 Generała Sylwestra Kaliskiego St., 00-908 Warsaw, Poland; marcin.wachowski@wat.edu.pl; 3 Faculty of Chemistry, Warsaw University of Technology, 3 Noakowskiego St., 00-664 Warsaw, Poland; rzurowski@ch.pw.edu.pl

**Keywords:** ceramic–intermetallic composites, pulse plasma sintering, NiAl, mechanical properties

## Abstract

The scientific goal of this paper is to study and explain the relationship between the microstructure of a ceramic–intermetallic composite fabricated by consolidating a mixture of Al_2_O_3_ and NiAl-Al_2_O_3_ using the PPS technique and its basic mechanical properties. Six series of composites were manufactured. The obtained samples differed in the sintering temperature and content of compo-powder. The base powders, compo-powder, and composites were investigated using SEM equipped with an EDS and XRD. Hardness tests and K_IC_ measurements were applied to estimate the mechanical properties of the fabricated composites. The wear resistance was evaluated using a “ball-on-disc” method. The results demonstrate that the density of the obtained composites increases with the increased temperature of the sintering. The content of NiAl + 20 wt.% Al_2_O_3_ did not have a determining effect on the hardness of the manufactured composites. The highest hardness, contacting 20.9 ± 0.8 GPa, was found for the composite series sintered at 1300 °C and 2.5 vol.% of compo-powder. The highest K_IC_ value from all the studied series equaled 8.13 ± 0.55 MPa·m^0.5^ and was also achieved for the series manufactured at 1300 °C (2.5 vol.% of compo-powder). The average friction coefficient during the ball-friction test with the Si_3_N_4_ ceramic counter-sample was between 0.8 and 0.95.

## 1. Introduction

Developing technologies requires the design of new materials and methods of their fabrication. In particular, composite materials are candidates for meeting expectations and possible applications in the medicine, automotive, and aerospace industries and for production cutting, machining tools, and many other purposes. The group of ceramic matrix composites linking ceramics with metals or intermetallic phases allows combining the dissimilar properties of various materials and creating new ones with a spectrum of properties. By the incorporation of various metal or intermetallic particles into a ceramic matrix, the mechanical and tribological properties can be significantly improved [1,2,3,4,5,6,7,8]. Magnetic or electric properties or thermal conductivity can be shaped as well [9,10]. For this reason, new custom-made ceramic–metal and ceramic–intermetallic composites have been intensively elaborated and investigated.

According to the state of knowledge, the properties of the composites can be effectively changed by the size and distribution of the incorporated phases. Ductile metal particles of micro sizes improve the fracture toughness of brittle ceramic matrices. The mechanisms for increasing the fracture toughness in such composites are well described in the literature [1,2,3]. For this reason, microparticles of Al, Cu, W, Mo, Ni, Ti, and Cr are used and combined with different ceramics [1,2,3,5,6]. For example, in composites of Al_2_O_3_-Al systems, a high degree of toughness and electrical conductivity have been achieved, which makes them candidates to be applied as internal combustion engine piston crowns, connecting rods, and turbine compressors [7]. Good mechanical and tribological properties of Al_2_O_3_-Mo composites are desired in applications as structural materials [8]. Moreover, metals as good heat conductors can also give higher thermal conductivity than ceramics. For example, such results were obtained with Al_2_O_3_-Ni where the size of the Ni particles was equal to approximately 1.4 µm [9] and with an Al_2_O_3_-Ag composite with Ag particles of a size equal to 2.7 µm [10].

The enhancement effects of nanoparticles on the mechanical properties of ceramic matrix composites is also well documented. For example, composites with Al_2_O_3_ or ZrO_2_ matrices containing nanoparticles (<100 nm) of metals Mo, Ni, W, Cu, and Al have been investigated in the literature [11,12,13]. Nanocomposites have very high hardness and improved fracture toughness in comparison to ceramics [2]. Such nanocomposites, e.g., Al_2_O_3_-Ni, are highly desired as protective coatings and cutting tools [13]. In addition, our own investigations of ceramic–metal nanocomposites of ZrO_2_-Ti and ZrO_2_-Ni systems have revealed the hardening effect of nanoparticles of metal or metal oxides [14,15,16,17,18]. For example, in ZrO_2_-Ti composites new phases from a Zr-Ti-O system were found, i.e., the very fine (100 nm) precipitation of titanium oxides and ZrTiO_2_ [18,19].

However, new materials that require more complex properties result from a combination of component functions. Many efforts are being devoted to designing such composites, which makes them suitable for advanced engineering applications. As a solution, micro–nano complex ceramic matrix composites have been elaborated. Such composites can combine more than two materials, such as two ceramics, with one or more metal or inter-metallic phases of various sizes and shapes. This means that, finally, the complex micro–nano structure of composites can be formed. Some such materials can represent hybrid materials. According to Ashby and Brecht, a hybrid material is defined as a combination of two or more materials in a predetermined geometry and scale, optimally serving a specific engineering purpose [20].

As a result, new multiphase materials with improved properties, such as yield strength, toughness, flexural strength, hardness, wear resistance, and other properties can be achieved. In recent years various multiphase composites have been obtained and investigated, for example ZrO_2_-Ti-Ni [21], Ni-Ti-ZrO_2_ [22,23], Al_2_O_3_ with ZrO_2_ and Ti(C,N) [24], Al_2_O_3_-ZrO_2_-Nb [25], Al_2_O_3_-ZrO_2_-CeO_2_ with the addition of Ni [26], as well as ZrO_2_ with Al_2_O_3_ and (Ti,W)C [27].

Also in ceramic–intermetallic systems, the improving of composite properties is achieved as a consequence of specific microstructures. For example, in Al_2_O_3_-NiAl composites the toughening effect is a result of crack deflection because of a weak Al_2_O_3_/NiAl interface and crack bridging by the interconnected, elongated NiAl flakes. Simultaneously, the high strength of the composites is because of their refined microstructures [28]. There are more reasons for designing ceramic–intermetallic composites. Intermetallic compounds are potential materials for high-temperature applications with superior oxidation and corrosion resistance [29].

Ceramic–metal as well as ceramic–intermetallic composites can be fabricated by various conventional powder processing and powder metallurgy techniques (PM). Advanced sintering techniques are also used. Among them are reactive hot pressing (RHP) [30], self-propagating high temperature synthesis (SHS) [31], combustion synthesis (CS) [32], spark plasma sintering (SPS) [33,34], and pulse plasma sintering (PPS) [35,36,37]. Different methods have various influences on the formation of multiphase composite materials. The distribution of phases, their sizes, new reaction phases, and porosity are shaped by adequate processing conditions. The application of the proper method and its parameters also offers the possibility to obtain gradient composites [38].

**Figure 1:**
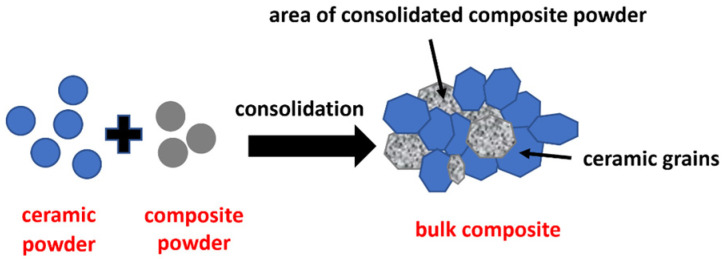
Schema of the microstructures of composites obtained by the consolidation of ceramic and composite powders.

In all of the techniques based on powder consolidation, mostly the powders are prepared separately before the composite fabrication powders are used. However, reinforcements can also be created by chemical reactions during the fabrication of composites as well as prepared in a preliminary process before the final consolidation. The second possibility for production of composites includes in the first step preparing composite powder. In the second step the obtained powder is mixed with ceramic powder and consolidated to prepare the final bulk composite material. Composite powder can be prepared from initial substrates such as ceramics and others which are made while processing compounds. Such a situation arises during the mixing of powders, such as two metals, which make new compounds as an intermetallic phase, together with ceramic powder. As a result, the composite powder consists of ceramics and an intermetallic phase. In the next step, the composite powder is mixed with ceramic powder and consolidated, making the ceramic–intermetallic bulk composite. This method of shaping the ceramic matrix composite with an intermetallic phase is presented in this paper. The microstructure of such a prepared composite is complex and consists of a ceramic matrix with an intermetallic phase distributed in it and ceramic particles trapped inside. In this case, it is possible to obtain a uniform distribution of constituents and then retain it in the final composites. Moreover, a complex microstructure is shaped. A schematic view of the expected microstructures of composites obtained by the consolidation of ceramic and composite powders is presented at Figure 1.

Ceramic–intermetallic composites (Al_2_O_3_-NiAl) are investigated in the present paper. The composite powder consisted of the two-phase NiAl, and Al_2_O_3_ was chosen as the reinforcement for the Al_2_O_3_ matrix. In the first step, by the process of milling (MA), the composite powder was produced using Ni and Al metal powders and Al_2_O_3_ powder. Through the reaction between Ni and Al, the NiAl is constituted trapped inside Al_2_O_3_ particles. Previous work has shown that the produced powder has a composite structure with fine Al_2_O_3_ particles uniformly distributed in the nanocrystal-like NiAl intermetallic matrix (crystallite phase 12–14 nm) [35]. Moreover, investigations have revealed that such composite powder can be well-consolidated by using a PPS method in which the powder is uniaxially pressed during heating by periodically generated electric pulses [36]. Because of the very short sintering time in the PPS method, bulk materials are formed very rapidly, which protects against a coarsening structure [36,37,38,39]. The very short sintering time was crucial for selecting the PPS method to consolidate the alumina and composite powder mixture. Although both PPS and the more widespread SPS are based on their operating principle of the conversion of electrical energy into internal energy according to Joule’s law, they are not equivalents. The PPS procedure is carried out under a high vacuum and has a high-voltage character. This means that, as opposed to low-voltage SPS sintering, the process is characterized by a significantly higher intensity due to the considerably higher current density flowing through the material for the duration of the pulse [39,40,41,42]. Previous experiments have revealed the high density, hardness and plasticity of NiAl- Al_2_O_3_ composite powder consolidated by this method. Moreover, after the process of consolidation of composite powder, bulk material is characterized by two phases, i.e., Al_2_O_3_ located within the NiAl matrix [35,43].

In previous research, Al_2_O_3_ matrix composites have been fabricated by the slip casting method, using NiAl-Al_2_O_3_ powder as the initial powder [44]. A composite powder consisting of NiAl + 30 wt.% Al_2_O_3_ has also been obtained by mechanical alloying of Al_2_O_3_, Al, and Ni powders. The composite powder mixed with Al_2_O_3_ powder after slip casting caused the formation of a high-density bulk composite [44]. After the sintering process of the samples in an air atmosphere the NiAl_2_O_4_ spinel phase appeared. Finally, a three-phase ceramic composite consisting of Al_2_O_3_, Ni, and NiAl_2_O_4_ was obtained. Important is the fact that in the area of Ni, fine Al_2_O_3_ particles remaining from the initial composite NiAl-Al_2_O_3_ powder were located, which makes the complex structure of the composite [44].

These results of preliminary experimental work are promising for further study of the consolidation of composite NiAl–Al_2_O_3_ powder with various initial contributions of ceramic and composite powders, which is the aim of the present paper.

The subject presented in this manuscript is novel. The literature data show that there are currently not many studies that use a pre-composite powder containing an intermetallic (NiAl) phase formed in the process of mechanical alloying (MA) from the starting substrate metals (Ni, Al) mixed with a ceramic powder (Al_2_O_3_). For this article, a study was conducted in which a powder containing intermetallic NiAl and 20 wt.% Al_2_O_3_ was prepared in the first stage. This powder was then mixed with a ceramic powder (Al_2_O_3_) and subjected to PPS consolidation to obtain the final solid ceramic matrix composite with an intermetallic phase. The scientific goal was to investigate and describe the relationship between the microstructure of the ceramic–intermetallic composite obtained by consolidating a mixture of ceramic (Al_2_O_3_) and pre-composite (NiAl-Al_2_O_3_) powders using the PPS method and its basic mechanical properties.

The achievement of this scientific goal, along with the presented conditions and the applied research methodology, will lead to the utilitarian goal, which is to create the basis for the design of ceramic–intermetallic composites.

## 2. Materials and Methods

### 2.1. Materials

The ceramic powder used in this research was α-Al_2_O_3_ (TAIMEI CHEMICALS Co., Ltd., Tokyo, Japan). According to the manufacturer’s specifications, the powder particle size was 100 ± 20 nm and had a spherical shape. In the investigations, Al and Ni were used as metallic powders. The Al powder (ABCR GmbH & Co. KG, Karlsruhe, Germany) had an average particle size of 44 μm. The Ni powder (ABCR GmbH & Co. KG, Karlsruhe, Germany) had a 3–7 μm average particle size. The theoretical density of the powders used was as follows: Al_2_O_3_—3.98 g/cm^3^, Ni—8.9 g/cm^3^, and Al—2.7 g/cm^3^. The initial powders had purities of: 99.7%—Al, 99.9%—Ni, and 99.99%—Al_2_O_3_. The information about particle size and purity was established based on the manufacturer’s data. The choice of raw powders was based on the size of the starting powders and their high purity.

### 2.2. Preparation of Specimens

The first step of the research was mechanical alloying to prepare a composite powder. A powder containing intermetallic NiAl and 20 wt.% Al_2_O_3_ was prepared. In the following, the powder is referred to as the compo-powder. Mechanical alloying has been successfully used for years to produce intermetallic materials and composites with an intermetallic phase matrix. A SPEX 8000D high-energy shaker ball mill (SPEX^®^ SamplePrep, Metuchen, NJ, USA) was used for milling a blend of Ni-50Al (at.%) elemental powders with the addition of 20 wt.% of Al_2_O_3_ powder. The milling procedures were performed under an argon atmosphere. An 8:1 ball-to-powder weight ratio was used. During MA, balls with diameters of 10 and 12 mm were applied. The balls used in the mechanical alloying were made of bearing steel balls.

Subsequently, the samples were formed from the prepared powder. The samples were made via the pulse plasma sintering method (PPS). Four operations were carried out during the PPS method: preparing the working atmosphere, setting the press pressure, sintering, and chamber discharge [43,44,45,46,47]. In the first step the required low vacuum level equal to 1 Pa and a high vacuum equal to 10^−3^ Pa were achieved. In the next step, the die was pressed with the force starting from 20 MPa and ending at 80 MPa. This step leads to the establishment of the pressure prevailing during the process. In the sintering stage, current pulses with specific parameters were applied [35]. At the final stage of the process, the chamber was discharged, and atmospheric pressure was established inside the chamber. The PPS process parameters applied for the investigated samples were designed based on earlier investigations and are presented in Table 1 [35,39]. The PPS process used allowed for the production of cylinder-shaped composites with a diameter of 10 mm and a height of 6–8 mm.

Six series of the composites were fabricated. The obtained samples differed in the sintering temperature and content of the compo-powder (NiAl + 20 wt.% Al_2_O_3_). Detailed compositions of each series are listed in Table 2.

### 2.3. Powder Density Measurement

The actual density of the obtained NiAl + 20 wt.% Al_2_O_3_ powder was estimated using a helium pycnometer (AccuPyc 1340 II, Micromeritics, Norcross, GA, USA). Density measurements were calculated using the ASTM D3766 standard [48]. A cylindrical measuring chamber with a volume of 2.781 cm^3^ was used during the study. During measurements, the chamber filling pressure with helium was 19.5 psig (0.13 MPa), the measurement accuracy was 0.03%, and the measurement repeatability was ±0.01%. The powder was analyzed in the helium with parameters of 700 cycles and 10 rinses.

### 2.4. Hydrostatic Method

The selected physical properties of the samples, such as relative density, soaking, and open porosity, were determined using the Archimedes method. Measurements were accomplished according to the European Standard EN623-2 [49]. In this experiment, samples were weighed in air and subsequently immersed in distilled water and boiled for 1 h. Afterward, the composites were weighed in distilled water and then in the air without removing water from the pores [49].

### 2.5. Phase Composition Analysis

X-ray diffractometry investigations of the powder after MA and specimens were performed on a Rigaku MiniFlex II diffractometer (Rigaku Corporation, Tokyo, Japan) working at 15 mA and 30 kV in a step-scanning mode with a counting time of 3 s and step size of 0.05° at diffraction angles 2θ ranging from 23° to 120°. The CuKα radiation (λ = 1.54178 Å) was used in the XRD analysis. The ICDDPDF-4 + 2020 X-ray standard database was used to interpret the results. The XRD phase composition analysis provides an opportunity to control the course of the pre-composite powder preparation process over time and the changes that occur in it during MA. Furthermore, it also allows potential alterations occurring in the composite during the manufacturing process of the final composite samples to be controlled. Although it provides a great amount of information, there is no lasting effect on the material under investigation.

### 2.6. Williamson–Hall Method

To estimate the mean crystallite size of the NiAl phase in the final milling product, the Williamson–Hall method was used. The Williamson–Hall analysis is a simplified integral breadth technique where both size-induced and strain-induced broadening are deconvoluted by assessing the peak width as a function of 2θ [50,51]. In the Williamson–Hall analysis, by plotting the FW (s) × cos θ on the *y*-axis against sin θ on the *x*-axis, we obtain the strain component from the slope and the grain or crystallite size component from the y-intercept [50,51,52]. Even though X-ray profile analysis is an average method, it still holds a necessary position for crystallite size determination after mechanical alloying.

### 2.7. Microscopic Observations

The microstructures of the raw Al_2_O_3_, Al, Ni, and NiAl + 20 wt.% Al_2_O_3_ powders and produced composites were observed using a JEOL JSM-6610 scanning electron microscope. Microstructure analyses were conducted to determine how the NiAl phase was distributed in the composites. The microstructures were observed using SE (secondary electrons) and BSE (back-scattered electrons) modes, under a voltage of 15 kV. Before observation, the samples were covered with a thin layer of carbon. For this purpose, a QUORUM Q150T ES sputtering device was applied (Headquarters, Laughton, UK).

To determine the chemical composition of the NiAl + 20 wt.% Al_2_O_3_ powder and produced composite, surface microanalyses were accomplished using an Oxford X-Max electro-dispersive spectrometer (EDS) (Oxford Instruments, Oxfordshire, UK).

### 2.8. Hardness Test

The Vickers hardness method was used to determine hardness. The measurements were accomplished using an HVS-30T hardness tester (Huatec Group Corporation, Beijing, China) on the surfaces prepared for metallographic observation. During the measurements, a load of 49.05 N and an applied load time of 10 s were used. To determine the hardness, the following formula (Equation (1)) was used:

(1) $$HV=0.1819\cdot \frac{F}{d}$$

In the formula, the established hardness *d* is the arithmetic mean of the diagonal lengths of the impression obtained after the removal of the load in mm, and *F* corresponds to the load applied to the specimen in N. Twelve measurements were made for each sample.

### 2.9. Fracture Toughness

The fracture toughness determination of the fabricated composites was carried out using the indentation technique. The Lankford equation (Equation (2)) was applied to determine the fracture toughness coefficient K_IC_ [53]:

(2) $$K_{IC}=0.0782\cdot \left(HV\cdot a^{0.5}\right)\cdot {\left(\frac{E}{HV}\right)}^{0.4}\cdot {\left(\frac{c}{a}\right)}^{-1.56}$$

where HV stands for Vickers hardness (GPa), a is a diagonal half-length of Vickers impression (m), E is Young’s modulus (GPa), and c stands for average crack length (m).

The Young’s modulus of the fabricated composite samples was estimated according to the rule of mixtures considering the proportion of components in the material. Young’s modulus values, for Al_2_O_3_ equal to 380 GPa [54] and for NiAl equal to 186 GPa [55], were taken as the initial values for the calculations. As a result, the Young’s modulus value used in the calculations for the composite with 2.5 wt.% pre-composite powder was 376 GPa, while for 5 wt.% it was 382 GPa.

The hardness and fracture toughness examinations provide basic information about the mechanical properties of the fabricated composite specimens with pre-composite powder and the influence of the sintering temperature on them. This allows the fabrication process parameters to be controlled in terms of the expected mechanical characteristics, which the finished samples are expected to meet too. Ultimately, the determination of fracture toughness using the indentation technique does not generate what is necessary for preparing test specimens with specified geometries. By knowing the basic mechanical properties of the specimens, further processing of the specimen can be determined followed by further analysis of the mechanical properties.

### 2.10. Wear Resistance Test

**Figure 2:**
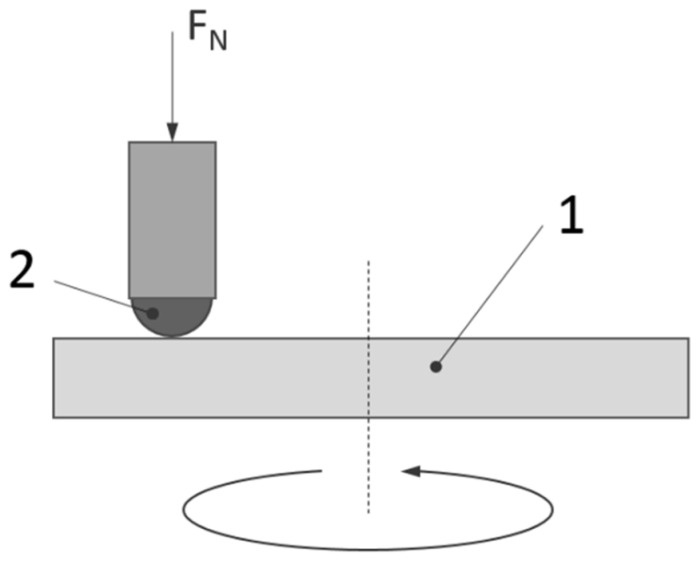
Scheme of the ball-on-disc method (1—sample, 2—tribometer head).

The wear resistance was evaluated by a “ball-on-disc” method, using a T-21 tribometer by ITEE, Radom, Poland. A sliding wear test was conducted without lubrication at constant room temperature conditions of 24 °C. A counter sample of Si_3_N_4_ with a diameter of 6.25 mm was used. Before the test, the surfaces of the sample and counter-sample were degreased in acetone. After each test, the sample and counter-sample surfaces were observed on a Nikon Eclipse LV150N light microscope (Nikon Metrology Inc., Brighton, MI, USA). The sliding wear test was carried out under a load of 10 N, on a radius of 5 mm, and with a linear speed of 0.1  m/s. A total of 5000 revolutions were performed, equivalent to 94.25 m total distance covered of the counter sample. The hardness value of the counter body Si_3_N_4_ was 78 HRC (1600 HV). The scheme of the ball-on-disc method is shown in Figure 2.

## 3. Results

**Figure 3:**
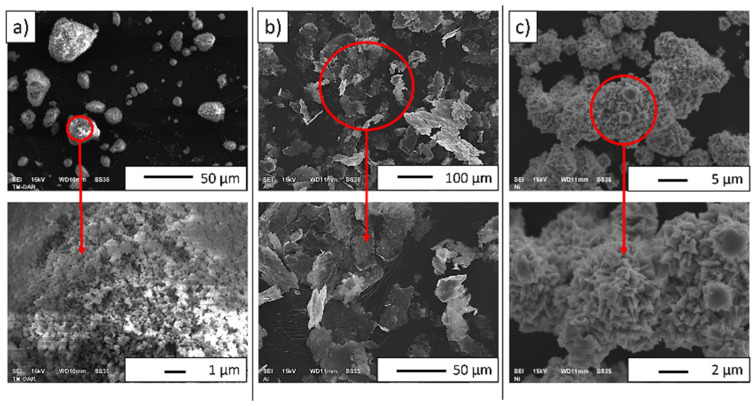
SEM images of powders: (**a**) alumina, (**b**) aluminum, and (**c**) nickel.

SEM images of the starting powders are provided in Figure 3. Based on SEM micrographs, it was found that the actual sizes of the powders were close to the manufacturer’s stated size. It was found that the alumina had a tendency to form agglomerations (Figure 3a). Moreover, it was observed that the Al_2_O_3_ powder (Figure 3a) had a regular shape. The aluminum particles demonstrated a flaky morphology (Figure 3b). The obtained micrographs show that the nickel powder was characterized by a cubic shape forming agglomerates (Figure 3c). Moreover, based on the observation of the nickel SEM images, it was found that the powder demonstrated numerous protrusions on the surface of its particles.

**Figure 4:**
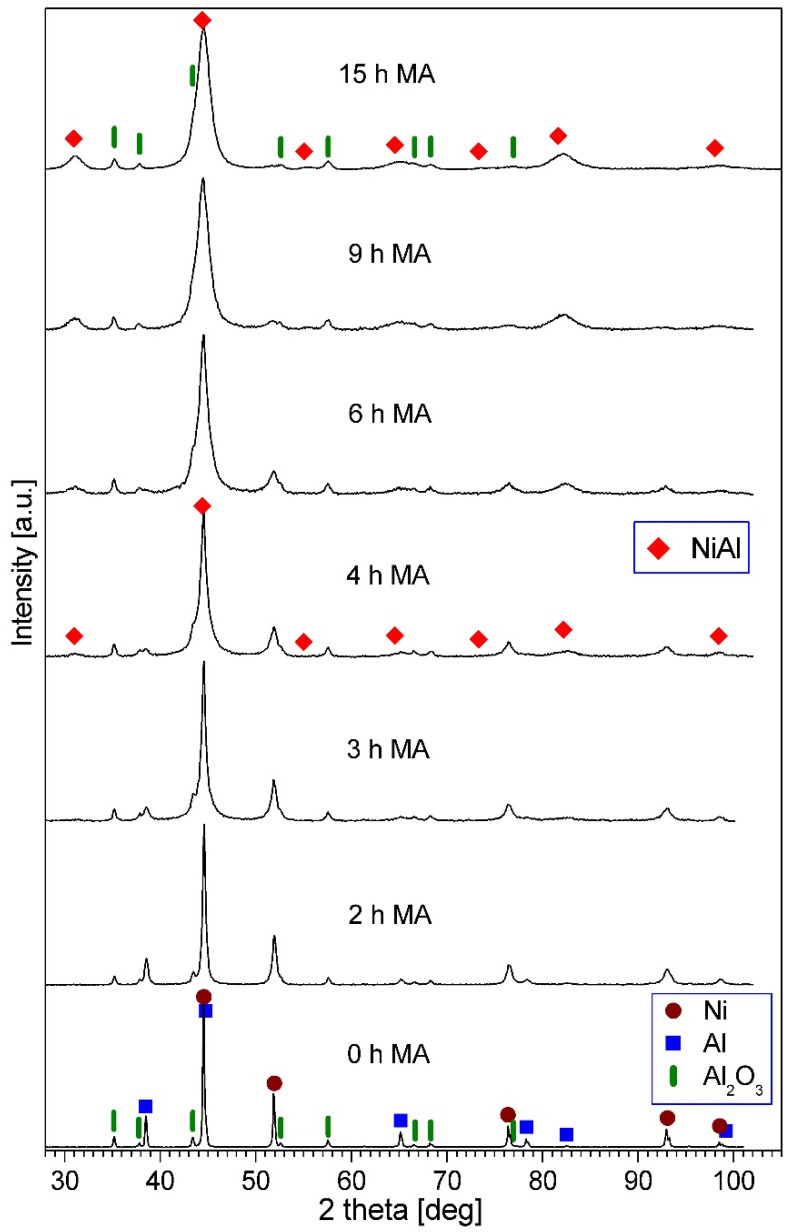
XRD patterns of the (Ni-50 at.% Al) + 20 wt.% Al_2_O_3_ powder blend milled for the times quoted.

The sequence of the XRD patterns of the (Ni-50 at.% Al) + 20 wt.% Al_2_O_3_ powder after various milling times is shown in Figure 4. In the patterns of powders for the early stage of milling, the peaks of Ni, Al, and Al_2_O_3_ are present. In the pattern of the 4 h milled powder, new peaks corresponding to a NiAl phase appear. The intensity of the Ni and Al diffraction lines decreases gradually with increasing milling time and finally they vanish. The intensity of the NiAl peaks increases with the progress of milling. The phase composition of the final product after 15 h of the process is the NiAl phase and Al_2_O_3_. The observed phase evolution, i.e., the gradual formation of an NiAl phase and disappearance of Al and Ni is similar to those reported earlier for the mechanical alloying of the Ni-50 at.% Al powder mixture [56] and for the milling of the Ni, Al, and Al_2_O_3_ mixtures used in this work, but containing 10% or 30% of corundum [35,43]. However, for the mixture containing 10% of Al_2_O_3_, the phase changes occurred faster, while for the mixture containing 30% of Al_2_O_3_, the phase changes occurred slower than in this work. The influence of the amount of reinforcing phase on the phase transformation rate and the NiAl phase formation during milling of Ni-Al-B powder mixtures has been reported [57]. It was observed that increasing the content of B in the mixture led to an increase in the milling time required for the formation of the NiAl phase and the disappearance of Al and Ni [57]. The impact of the reinforcing phase content on the phase changes during milling has also been observed in the case of Fe-Al-B powder mixtures [58]. The formation process of the NiAl intermetallic phase is consistent with the phase equilibrium diagram for this system [59].

**Figure 5:**
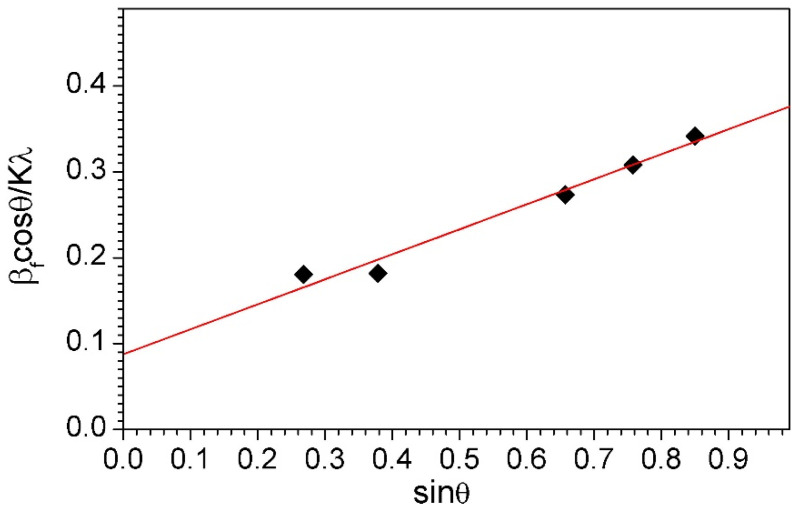
Williamson–Hall plot for the NiAl phase in the powder after 15 h of milling (final milling product).

The mean crystallite size of the NiAl phase after 6 h and 9 h of milling was estimated by the Williamson–Hall method and amounted to 21 nm and 14 nm, respectively. The mean crystallite size of this phase in the final product of milling was 11 nm and the estimated mean lattice strain was 0.01%. The Williamson–Hall plot for the NiAl phase in the powder after 15 h of milling (final milling product) is shown in Figure 5.

**Figure 6:**
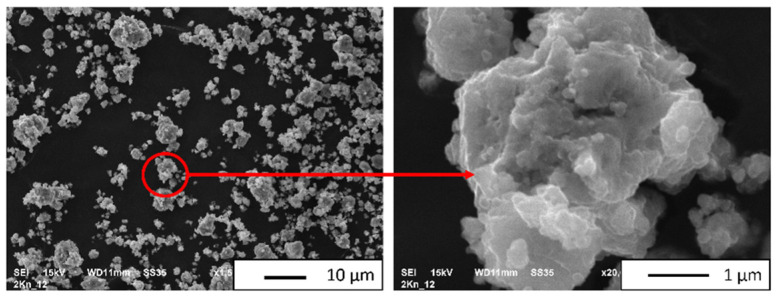
SEM image (SE mode) of powder after MA.

The density of the NiAl-20% Al_2_O_3_ powder, measured using a helium pycnometer, was 5.1858 g/cm^3^, which is consistent with previous measurements [38]. Figure 6 shows the microstructure of the NiAl-20% Al_2_O_3_ powder obtained after 15 h of milling (final milling product). Observations of the powder revealed that it consisted of agglomerates. It was found that the single particles of the NiAl-20% Al_2_O_3_ powder are almost spherical. The SEM observations reveal that the NiAl-20% Al_2_O_3_ powder particle sizes range from below 0.4 µm up to 11 µm. The obtained values are close to the powder size of NiAl + 30 wt.% Al_2_O_3_ powder after mechanical alloying for 18 h, which we obtained in previous work [43].

**Figure 7:**
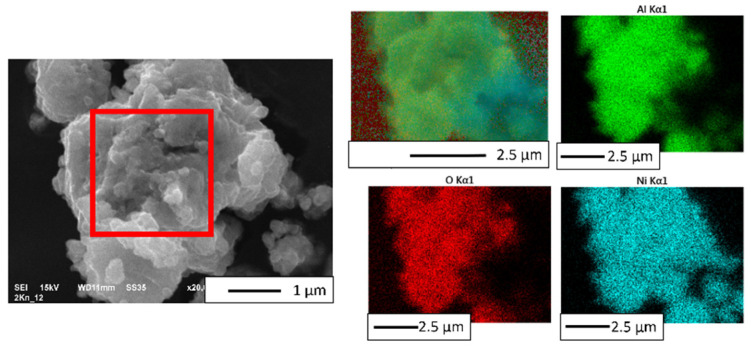
EDS image of powder after MA.

The chemical elemental distribution map of the powder after MA is shown in Figure 7. Analysis of EDS (Figure 7) revealed that only oxygen, aluminum, and nickel were found. A quantitative analysis of the recorded EDS spectra (area in Figure 7) is demonstrated in Table 3. Based on the values obtained, it was found that the obtained powder contained 36.26% by weight of aluminum, 13.50% by weight of oxygen, and 50.24% by weight of nickel. The obtained values indicate an even distribution of components during mechanical alloying. The obtained values of the NiAl-20% Al_2_O_3_ powder composition after mechanical alloying are close to the values obtained for the pre-composite powder in previous work [43].

The physical properties of the obtained samples are shown in Table 4. The obtained results indicate that the density of the obtained composites increased with increased sintering temperatures, which is consistent with the literature data [35,39]. The lowest density values were obtained in the case of Series I and IV sintered at 1200 °C (Series I—92.24%, Series IV—93.03%). The results showed that using a temperature of 1200 °C does not allow for obtaining high-density composites. Increasing the temperature by 100 °C ensures obtaining composites with a density of 99%. The formation of the samples at 1300 °C and 1400 °C gave a relative density value for all series >99%. The open porosity and soaking of Series II, III, V, and VI were close to zero.

**Figure 8:**
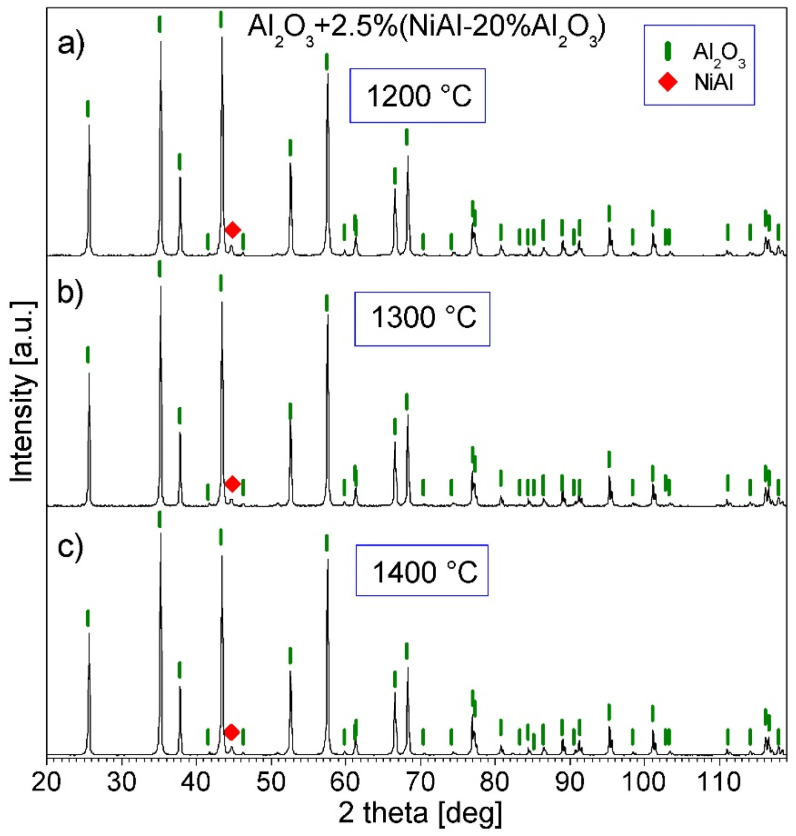
XRD patterns of the Al_2_O_3_ + 2.5% (NiAl + 20Al_2_O_3_) powder consolidated by PPS: (**a**) at 1200 °C, (**b**) at 1300 °C, and (**c**) at 1400 °C (Series I–III).

**Figure 9:**
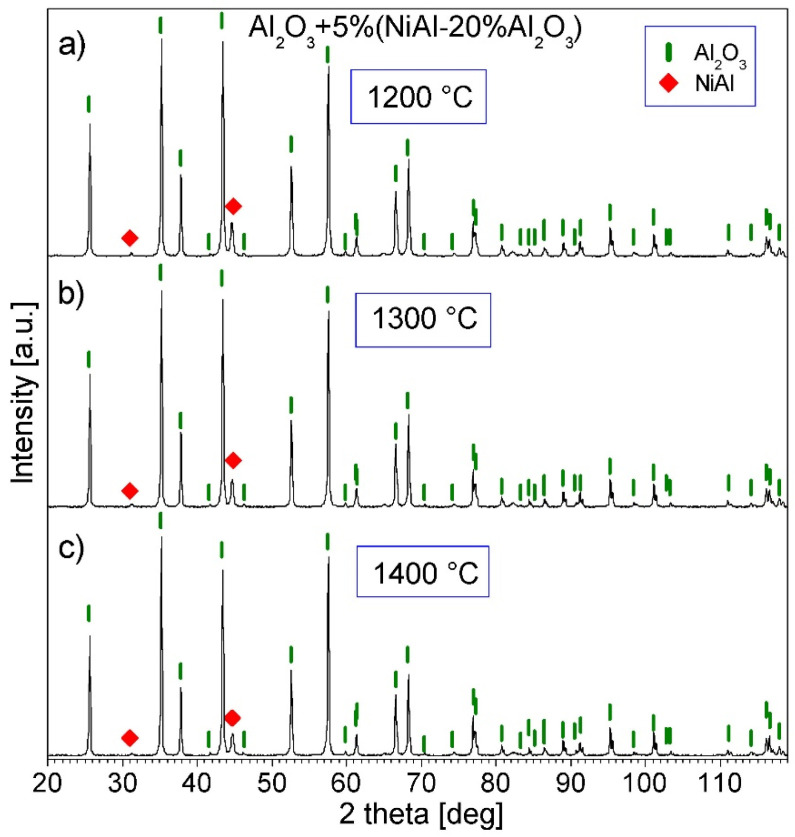
XRD patterns of the Al_2_O_3_ + 5% (NiAl + 20Al_2_O_3_) powder consolidated by PPS: (**a**) at 1200 °C, (**b**) at 1300 °C, and (**c**) at 1400 °C (Series IV–VI).

Figure 8 and Figure 9 show the XRD patterns of Series I–VI. Only peaks of the Al_2_O_3_ and NiAl phases are present in the patterns, which shows that no new phases were created during the PPS consolidation. The (100) diffraction line of the NiAl phase is better visible for Series IV–VI, since the amount of this phase in these samples is greater than in the others.

**Figure 10:**
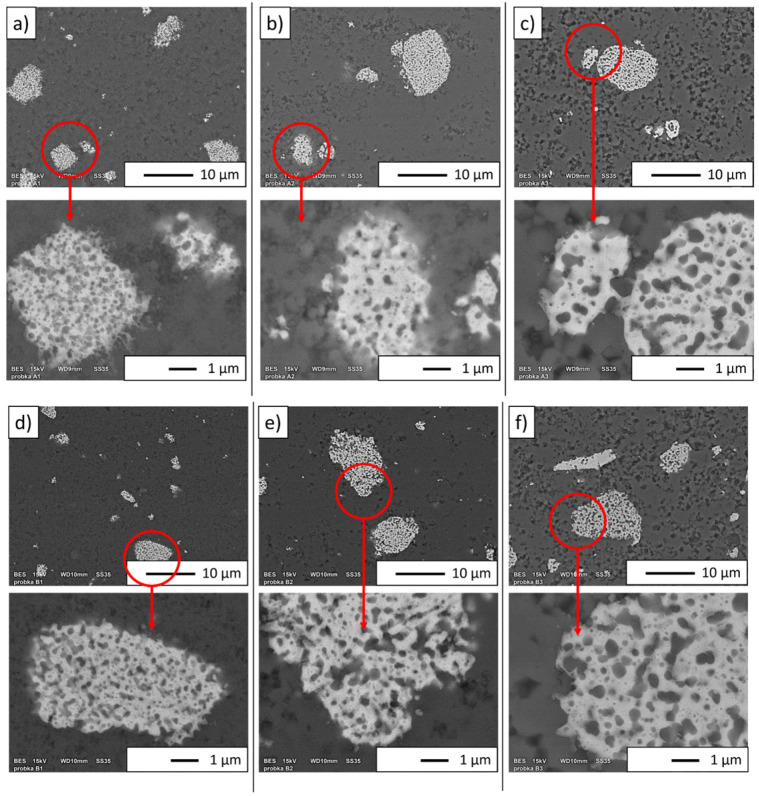
SEM images of the obtained samples: (**a**) Series I, (**b**) Series II, (**c**) Series III, (**d**) Series IV, (**e**) Series V, and (**f**) Series VI.

The SEM images of the obtained samples are shown in Figure 10. The red-marked areas correspond to magnified areas. In the micrographs obtained in BSE mode, the NiAl phase is demonstrated as light grey areas, while the Al_2_O_3_ phase is shown as dark grey areas (Figure 10). Observations showed that large and small particles of the NiAl phase were present in each produced series.

**Figure 11:**
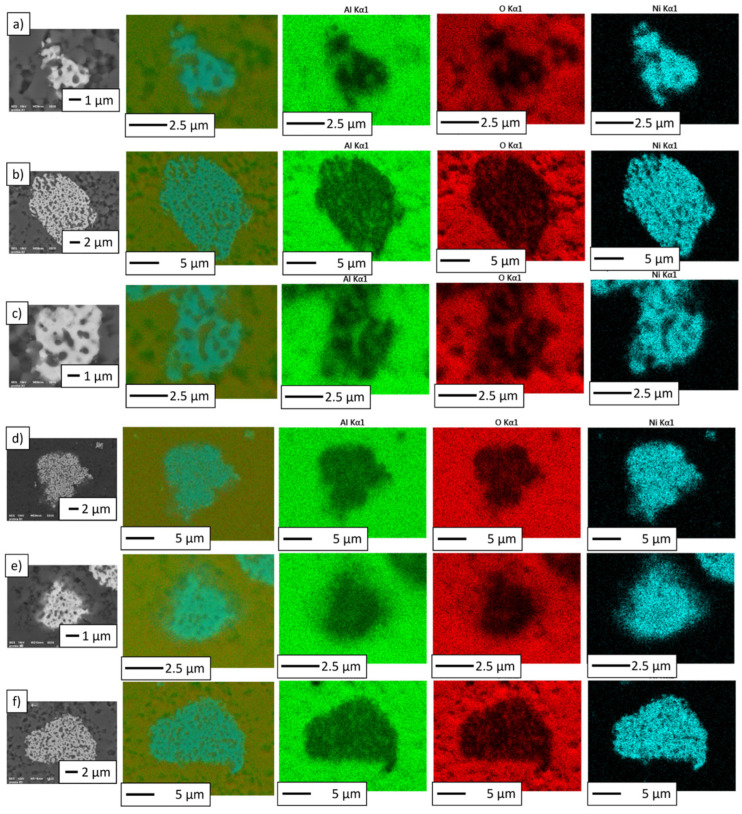
Distribution of elements on the surface of composites: (**a**) Series I, (**b**) Series II, (**c**) Series III, (**d**) Series IV, (**e**) Series V, and (**f**) Series VI.

A map of the distribution of elements for samples is presented in Figure 11. The distribution of elements measured by EDS microanalysis revealed that the obtained composites were formed of aluminum, oxygen, and nickel.

**Figure 12:**
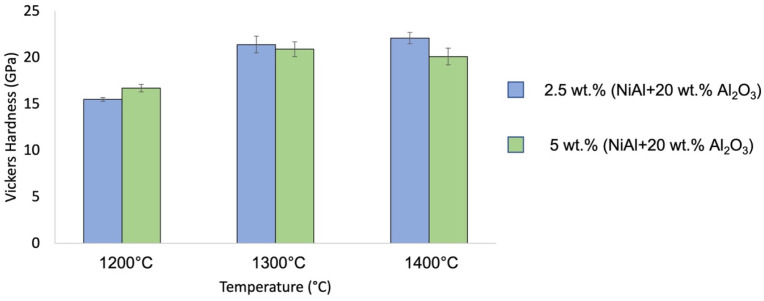
Vickers hardness of obtained samples.

The results of the Vickers hardness measurements are shown in Figure 12. The analysis of the averaged hardness values for the samples formed with 2.5 wt.% pre-composite powder revealed a close correlation between the results obtained and the sintering temperature applied. For higher sintering temperatures, a higher average hardness of the composite was achieved. Accordingly, the Series I sintered at 1200 °C was characterized by the lowest average hardness of 15.5 ± 0.2 GPa. Meanwhile, the highest hardness was measured for the Series III samples sintered at 1400 °C and was equal to 22.1 ± 0.6 GPa. For Series II, sintered at the intermediate temperature of 1300 °C, the measured hardness amounted to 21.4 ± 0.9 GPa.

For specimens with 5 wt.% pre-composite powder in their structure, the lowest hardness value, equal to 16.7 ± 0.4 GPa, characterized the Series IV specimens sintered at 1200 °C, the lowest among the examined temperatures. Simultaneously, the hardness obtained for this series was higher than for the corresponding one with a reduced amount of pre-composite powder in the structure. The highest hardness, reaching 20.9 ± 0.8 GPa, was estimated for Series II sintered at 1300 °C. However, a gentle decrease in hardness was observed as the sintering temperature increased. For Series VI, where the sintering process was carried out at 1400 °C, the hardness amounted to 20.1 ± 0.9 GPa. The higher proportion of pre-composite powder in the material structure during sintering at 1300 °C and 1400 °C led to a decrease in the hardness of the produced composites with respect to the analogous samples with a lower proportion of compo-powder (NiAl + 20 wt.% Al_2_O_3_).

For both composition types, a correlation between hardness and temperature of the process and therefore indirectly also the compaction of the sample is visible. The most pronounced differences can be seen between specimens sintered at 1200 °C (Series I and Series IV) and the remaining series. These samples were characterized by the lowest densification after the sintering process, which was reflected in their reduced mechanical properties. When sintered at higher temperatures, for which the relative density exceeded 99%, the differences in hardness are mainly dictated by the contribution of the pre-composite powder to the composite structure. Increasing its content from 2.5 wt.% to 5 wt. % led to a slight decrease in the hardness of the material. The hardness values obtained for the analyzed composites stand in good correlation with both previous research results and the available literature data. The hardness of both groups of the composite samples reached high values, similar to those of pure Al_2_O_3_ sintered under identical conditions [39]. Similar results were obtained for pure Al_2_O_3_ in the work of Yuan et al. [60], where an enhancement of Al_2_O_3_ hardness was observed with increased sintering temperatures. Both the work of Sun et al. [61] and Ouyang et al. [62] reported hardness for Al_2_O_3_ ceramic samples exceeding 20 GPa. This indicates that the addition of pre-composite powder (NiAl + 20 wt.% Al_2_O_3_) did not have a determining effect on the hardness of the fabricated composites.

The fracture toughness results for all of the obtained series are presented in Table 5. Regarding the series containing 2.5 wt.% of the pre-composite powder in the composite structure, the highest K_IC_ value of 8.13 ± 0.55 MPa·m^0.5^ was achieved for Series II manufactured at 1300 °C. However, the value obtained for Series III sintered at 1400 °C was only slightly lower with a K_IC_ equal to 7.42 ± 0.75 MPa·m^0.5^. The variation between the series was only 9.5%. The lowest fracture toughness, on the other hand, characterized Series I specimens after the process was carried out at 1200 °C, with a K_IC_ value equaling 6.75 ± 0.45 MPa·m^0.5^.

Meanwhile, in the samples with 5 wt.% contributions of the pre-composite powder to the structure, the highest value of the fracture toughness coefficient applied to Series VI sintered in 1400 °C with a K_IC_ equal to 8.39 ± 0.75 MPa·m^0.5^. The maximum achieved value was marginally higher compared to the series with a lower amount of the powder (NiAl + 20 wt.% Al_2_O_3_). The K_IC_ value for the Series V samples sintered at 1300 °C amounted to 6.73 ± 0.73 MPa·m^0.5^. The variation between the two series amounted to 25%. Similarly to the earlier group of series, the lowest fracture toughness value was exhibited by the Series IV samples following the process at 1200 °C with a K_IC_ of 5.84 ± 0.35 MPa·m^0.5^. An increasing trend in fracture toughness with rising sintering temperatures is noticeable for this series. This was not, however, apparent for the samples with a lower proportion of compo-powder (NiAl + 20 wt.% Al_2_O_3_).

The results achieved for both groups of the composite series follow the trend seen in previous studies. Both the samples with 2.5 wt.% and 5 wt.% pre-composite powder showed higher fracture toughness than those with pure Al_2_O_3_ [63]. At the same time, the differences in the proportion of the pre-composite phase were sufficiently small to ensure that, as in our prior studies with the different pre-composite powder compositions, the variations between the values are comparable [43].

**Figure 13:**
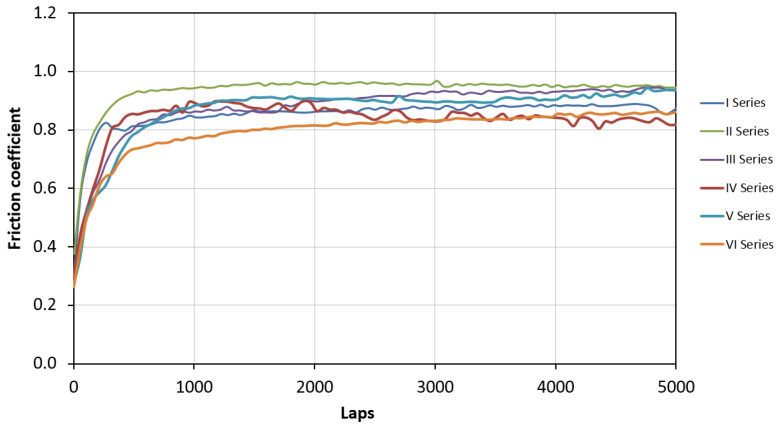
The course of changes in the friction coefficient during the wear resistance test.

In the initial stage of the wear test, due to the small contact area resulting from an initial mismatch of working surfaces, the recorded coefficient of friction was approx. 0.3. After breaking in the tribological system, requiring the counter-sample to cover a distance of approx. 500 revolutions, the friction coefficient stabilized (Figure 13). In the later part of the study, no sudden changes in the friction coefficient were observed.

**Figure 14:**
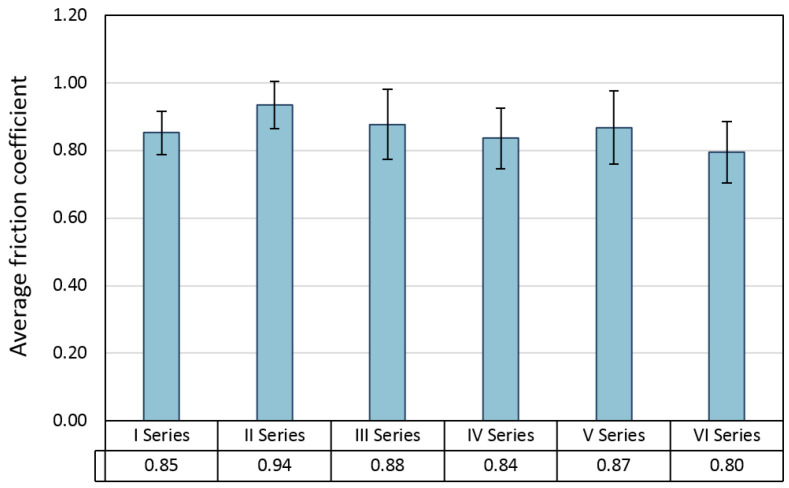
Average friction coefficient measured during the wear resistance test.

**Figure 15:**
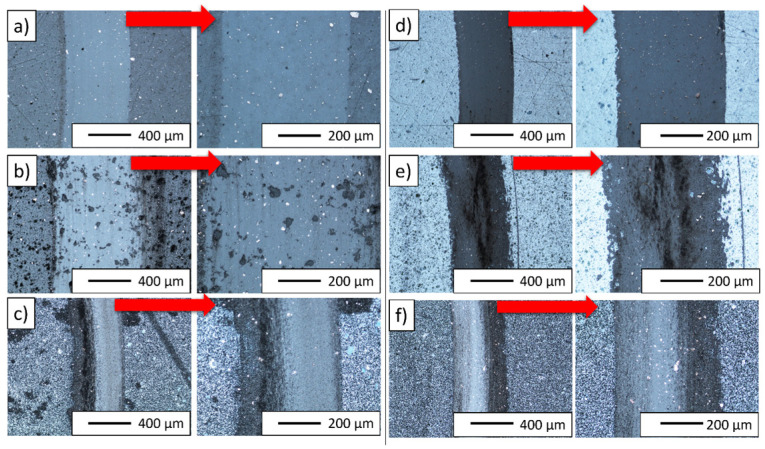
Wear tracks after ball-on-disc tests of composites: (**a**) Series I, (**b**) Series II, (**c**) Series III, (**d**) Series IV, (**e**) Series V, and (**f**) Series VI.

The average friction coefficient during the ball-friction test with the Si_3_N_4_ ceramic counter-sample was between 0.8 and 0.95 (Figure 14). In the case of both series of samples, containing 2.5% and 5% of the composite powder, a similar course of changes in the average friction coefficient depending on the sintering temperature was observed. In both cases, the value of the coefficient of friction for the sample sintered at 1300 °C was the highest (approx. 0.94 for 2.5% compo powder and 0.87 for 5% compo powder). The results of the friction coefficient relates to the width of the wear marks formed on the surface of the samples. In both cases, the abrasion widths on the samples produced at 1300 °C were the largest within the series (Figure 15). The friction coefficient values obtained for the tested samples are higher than those for pure Al_2_O_3_ ceramic samples sintered at 1300 °C with the SPS method in the study by Kumar et al. The CoF values they obtained varied between 0.35 and 0.4 [64].

Wear tracks after the ball-on-disc tests of composites for all series are shown in Figure 15. From the observation of the light microscope, it is concluded that all tested samples show typical abrasive wear. No substrate cracks were observed after the ball-on-disc test. It was found that the resulting abrasions have a low degree of development surface, without the occurrence of wear products in the abrasion track. It can be assumed that the observed widths of abrasions are largely due to the wear of the counter-sample ball of silicon nitride (Si_3_N_4_). Regular abrasion of the counter-sample in the case of materials characterized by very high resistance to wear by friction leads to an increase in the contact surface of the sample and counter-sample and a decrease in stresses in the area of the friction junction. The resulting traces of abrasion correspond more to the geometry of the contact than to the actual areas where the loss of the base material occurs.

## 4. Conclusions

This work presents the results of the application of the PPS method to produce bulk composite materials from the Al_2_O_3_–compo-powder (NiAl + 20 wt.% Al_2_O_3_) system. The composite compo-powder consisting of intermetallic NiAl and alumina phases was prepared by mechanical alloying. As initial powders, nickel, aluminum, and alumina were used. In the research, six different series with different sintering temperatures and compo-powder contents were produced and investigated. The performed experiments allowed description of the influence of the sintering temperature and content of the NiAl-Al_2_O_3_ powder on the sintering process and final properties of the final composites. The results revealed that Al_2_O_3_–compo-powder (NiAl + 20 wt.% Al_2_O_3_) composites can be fabricated using the PPS technique. According to the results obtained using the Williamson–Hall method, a time of 15 h of compo-powder milling was optimal for the final milling product. The results indicate that the density of the obtained composites increases with increases in sintering temperature. The content of the compo-powder did not have a determining effect on the hardness of the fabricated composites. The highest hardness, reaching 20.9 ± 0.8 GPa, was revealed for the composite series sintered at 1300 °C and with 2.5 vol.% of compo-powder.

Moreover, the same composite series (1300 °C and 2.5 vol.% of compo-powder) were characterized by the highest wear resistance, equal to 0.94 of the average friction coefficient. The highest K_IC_ value (8.13 ± 0.55 MPa·m^0.5^) from all the investigated series was also achieved for the Series II manufactured at 1300 °C (2.5 vol.% of compo-powder). The composite from this series was characterized by the highest achieved density results (99.80% ± 0.04%), lowest porosity (0.03% ± 0.01%), and soaking below 0.01%. The results give essential knowledge about the consolidation parameters and final properties of hybrid composites obtained by PPS with various initial contributions of mechanical-alloyed compo-powder and alumina and different sintering parameters.

## Figures and Tables

**Table 1:** The pulse plasma sintering process parameters for the specimens fabricated.

PPS Process Parameter	Stored Energy	Electro-Pulse Repetition	Voltage	Sintering Temperature	Heating Rate	Sintering Time	Load
	[kJ]	[s]	[kV]	[°C]	[°C/min]	[s]	[MPa]
Composite	2.77	1.3	4.3	1200, 1300, 1400	250	180	25–80

**Table 2:** Detailed compositions of each series.

Series	Type of Compo-Powder	Sintering Temperature	Content of Compo-Powder	Content of Al_2_O_3_
Series I	NiAl + 20 wt.% Al_2_O_3_	1200 °C	2.5 wt.% with respect to the amount of ceramic	97.5 wt.%
Series II		1300 °C		
Series III		1400 °C		
Series IV		1200 °C	5 wt.% with respect to the amount of ceramic	95 wt.%
Series V		1300 °C		
Series VI		1400 °C		

**Table 3:** Chemical composition of a characteristic area of the powder after MA. The measurement area is shown in Figure 7.

Measurement area is shown in Figure 7	Chemical Composition					
	O		Al		Ni	
	Weight%	Atomic %	Weight%	Atomic %	Weight%	Atomic %
	13.50 ± 0.12	27.73 ± 0.21	36.26 ± 0.17	44.15 ± 0.12	50.24 ±0.21	28.12 ± 0.09

**Table 4:** Selected physical properties of the samples.

Samples	Relative Density	Open Porosity	Soaking
	[%]	[%]	[%]
Series I—Al_2_O_3_ + 2.5 vol.% of compo-powder (NiAl + 20 wt.% Al_2_O_3_) sintered at temperature 1200 °C	92.24 ± 0.03	3.88 ± 0.11	1.04 ± 0.09
Series II—Al_2_O_3_+ 2.5 vol.% of compo-powder (NiAl + 20 wt.% Al_2_O_3_) sintered at temperature 1300 °C	99.80 ± 0.04	0.03 ± 0.01	<0.01
Series III—Al_2_O_3_+ 2.5 vol.% of compo-powder (NiAl + 20 wt.% Al_2_O_3_) sintered at temperature 1400 °C	99.85 ± 0.02	0.02 ± 0.01	<0.01
Series IV—Al_2_O_3_+ 5 vol.% of compo-powder (NiAl + 20 wt.% Al_2_O_3_) sintered at temperature 1200 °C	93.03 ± 0.02	3.79 ± 0.24	1.00 ± 0.08
Series V—Al_2_O_3_+ 5 vol.% of compo-powder (NiAl + 20 wt.% Al_2_O_3_) sintered at temperature 1300 °C	99.78 ± 0.04	0.03 ± 0.01	<0.01
Series VI—Al_2_O_3_+ 5 vol.% of compo-powder (NiAl + 20 wt.% Al_2_O_3_) sintered at temperature 1400 °C	99.85 ± 0.01	0.02± 0.01	<0.01

**Table 5:** KIC values for the obtained samples.

Samples	Composition	Sintering Temperature(°C)	K_IC_(MPa·m^0.5^)
Series I	Al_2_O_3_ + 2.5 vol.% of compo-powder (NiAl + 20 wt.% Al_2_O_3_)	1200	6.75 ± 0.45
Series II		1300	8.13 ± 0.55
Series III		1400	7.42 ± 0.75
Series IV	Al_2_O_3_ + 5 vol.% of compo-powder (NiAl + 20 wt.% Al_2_O_3_)	1200	5.84 ± 0.35
Series V		1300	6.72 ± 0.73
Series VI		1400	8.39 ± 0.75

## Data Availability

Data sharing not applicable.
